# Response of UK interventional radiologists to the COVID-19 pandemic – survey findings

**DOI:** 10.1186/s42155-020-00133-2

**Published:** 2020-06-26

**Authors:** Sammy Rostampour, Trevor Cleveland, Hilary White, Philip Haslam, Ian McCafferty, Mo Hamady

**Affiliations:** 1 Imperial College Healthcare NHS Trust, London, UK; 2 Sheffield Teaching Hospitals NHS Foundation Trust, Sheffield, UK; 3 Musgrove Park Hospital, Taunton, UK; 4 Newcastle Upon Tyne Hospitals NHS Foundation Trust, Newcastle upon Tyne, UK; 5 University Hospitals Birmingham NHS Foundation Trust, Birmingham, UK

**Keywords:** COVID-19, Interventional radiology, Survey, Endovascular

## Abstract

**Background:**

The COVID-19 pandemic has had an unprecedented effect upon the National Health Service (NHS). Like other specialties, Interventional Radiology (IR) rapidly adapted to the evolving situation. Members of BSIR were surveyed to obtain a snapshot of the experiences of UK IRs in response to COVID-19.

An electronic survey was compiled using Google Forms, approved by the BSIR Council Officers and distributed to BSIR members by email on 18 ^th^ April 2020. A total of 228 responses were received. The survey was open for a 14-day period and the data analysed in Microsoft Excel 365. The response rate was 29% (228/800).

**Results:**

Two thirds of respondents work in a Tertiary unit and 33% deliver IR in a District Hospital. 84% have a day-case facility. After the COVID-19 crisis, 81% of respondents were able to maintain 24–7 On-call service. 59% of respondents had been required change their day to day practice to allow the on-call service to continue. 55% of respondents were involved in providing a central line service. Of those questioned, 91% continued to offer endovascular services, 98% genitourinary and 92% hepatobiliary services, although a degree of service reduction was described. 38% have provided IR trainees with additional training material during this pandemic.

**Conclusions:**

This survey has confirmed that the responses of UK IR departments to the COVID-19 crisis have ensured vital on-call and urgent services have continued, including ongoing availability of most IR sub-specialties. Availability of a day case facility has possibly influenced the positive response.

## Background

The global COVID-19 pandemic has affected our professional and personal lives in an unprecedented way. The UK National Health Service (NHS) was required to rapidly adapt to the developing crisis. At the time of writing, the UK had recorded the fourth highest national number of cases in the world (more than 215,000 active cases) and over 32,000 deaths from novel Coronavirus infection reported (GOV.UK 2020). NHS hospitals have made swift, extensive changes to routine service delivery in response to the pandemic, including cessation of many elective services, minimizing face-to-face interactions to those considered urgent, whilst simultaneously accommodating a surge in patients requiring urgent respiratory support.

In line with recommendations outlined by the British Society of Interventional Radiologists, Interventional Radiology (IR) departments have responded quickly by suspending elective procedures whilst attempting to maintain a robust service for emergent and urgent cases (BSIR 2020). The COVID-19 crisis has also significantly affected IR training with some trainees being redeployed to diagnostic radiology or to other clinical areas, with complete cessation of IR training activity for some.

In order to obtain a snapshot of the responses of UK Interventional Radiologists, members of the British Society of Interventional Radiology (BSIR) were surveyed about their experiences during the peak time COVID-19 pandemic.

## Method

An electronic survey was compiled using Google Forms, approved by the BSIR Council Officers and distributed to BSIR members by email on 18th April 2020. A total of 228 responses were received. The survey was open for a 14-day period and the data analysed in Microsoft Excel 365. Around 800 active BSIR members are registered giving an approximate response rate of 29%.

## Results

**Fig. 1:**
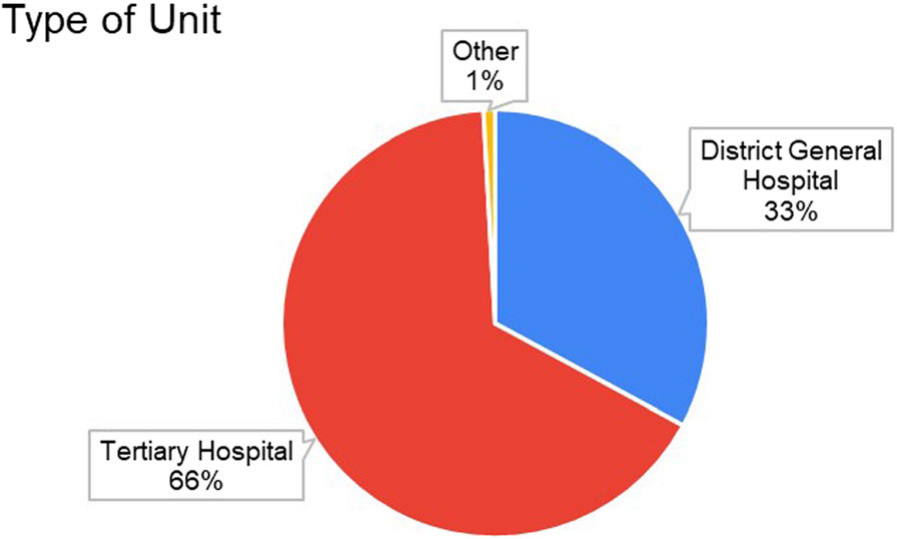
Type of IR unit of survey responders

**Fig. 2:**
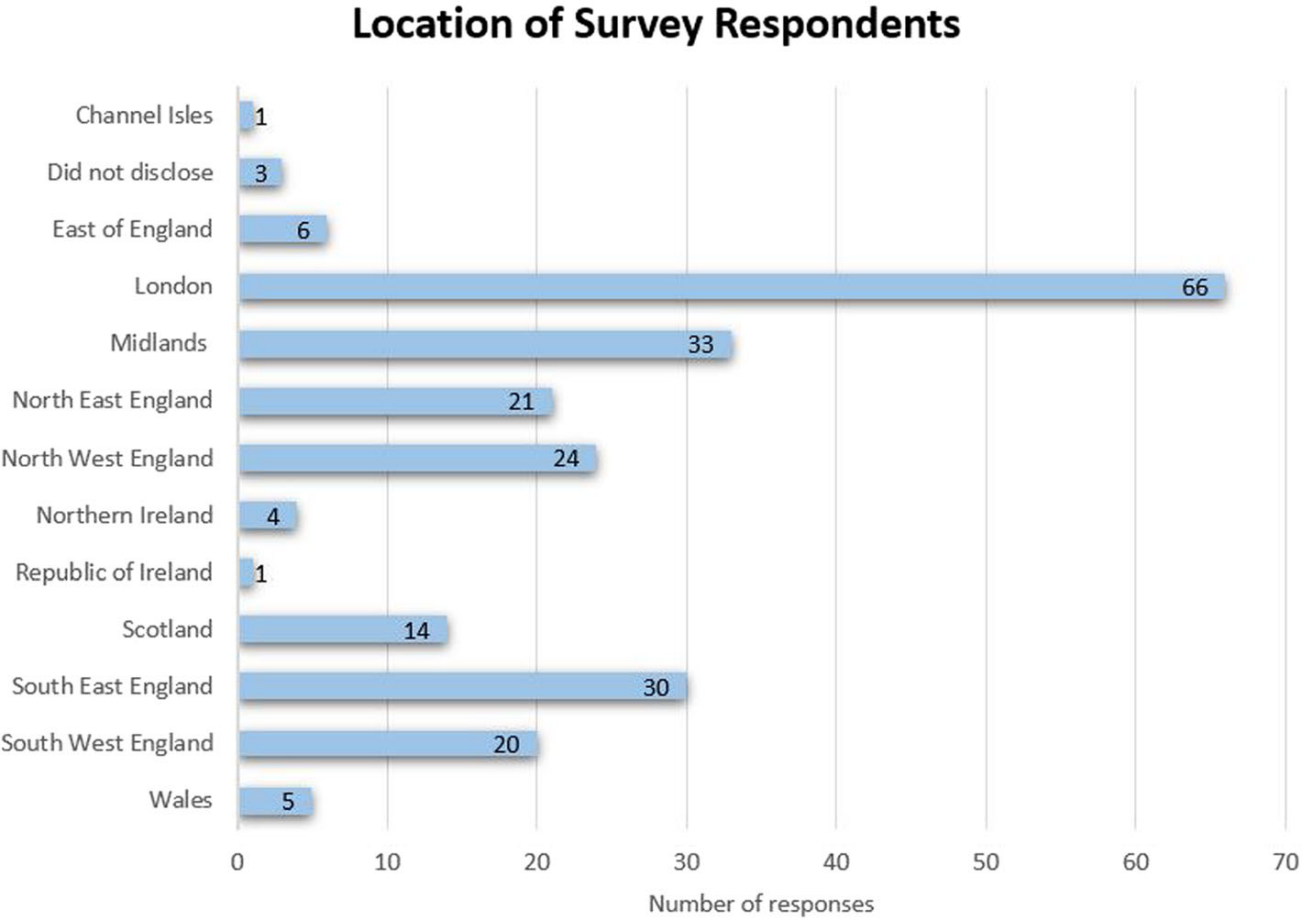
Location of survey respondents

**Fig. 3:**
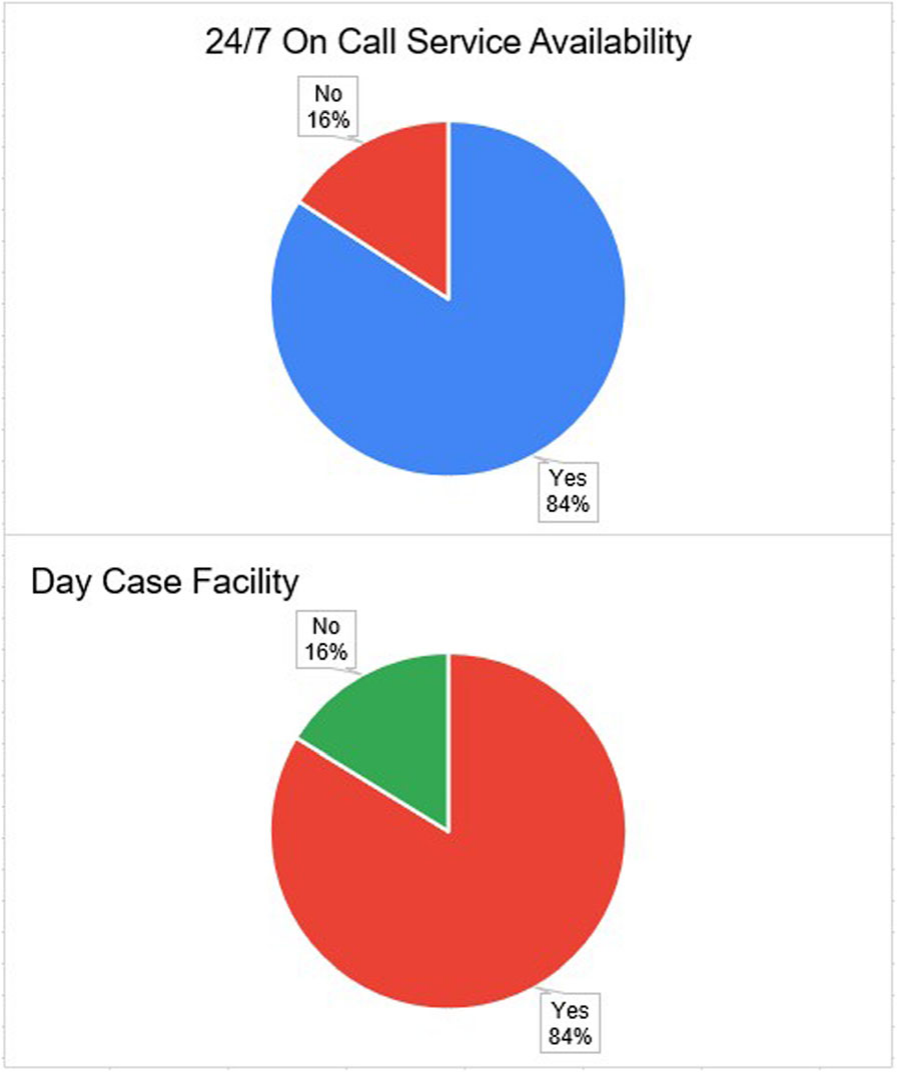
Availability of on call service and day case facility

Two thirds of respondents (66%) work in a Tertiary referral centre with 33% delivering IR in a District General Hospital (Fig. 1). Responses were received from a wide geographic distribution within the United Kingdom and Republic of Ireland. The largest responses were from London (29%), Midlands (14% and South East 13% (Fig. 2). Prior to the onset of the COVID-19 pandemic most institutions delivered Vascular IR (92%), Trauma IR (68%), Interventional Oncology (72%), Hepatobiliary (HPB) (87%) and Genitourinary (94%) services. The majority (84%) of those surveyed were active in the delivery of an Interventional On-call service, 24 h per day, and similar percentage (84%) have a day-case facility in their hospital (Fig. 3).

**Fig. 4:**
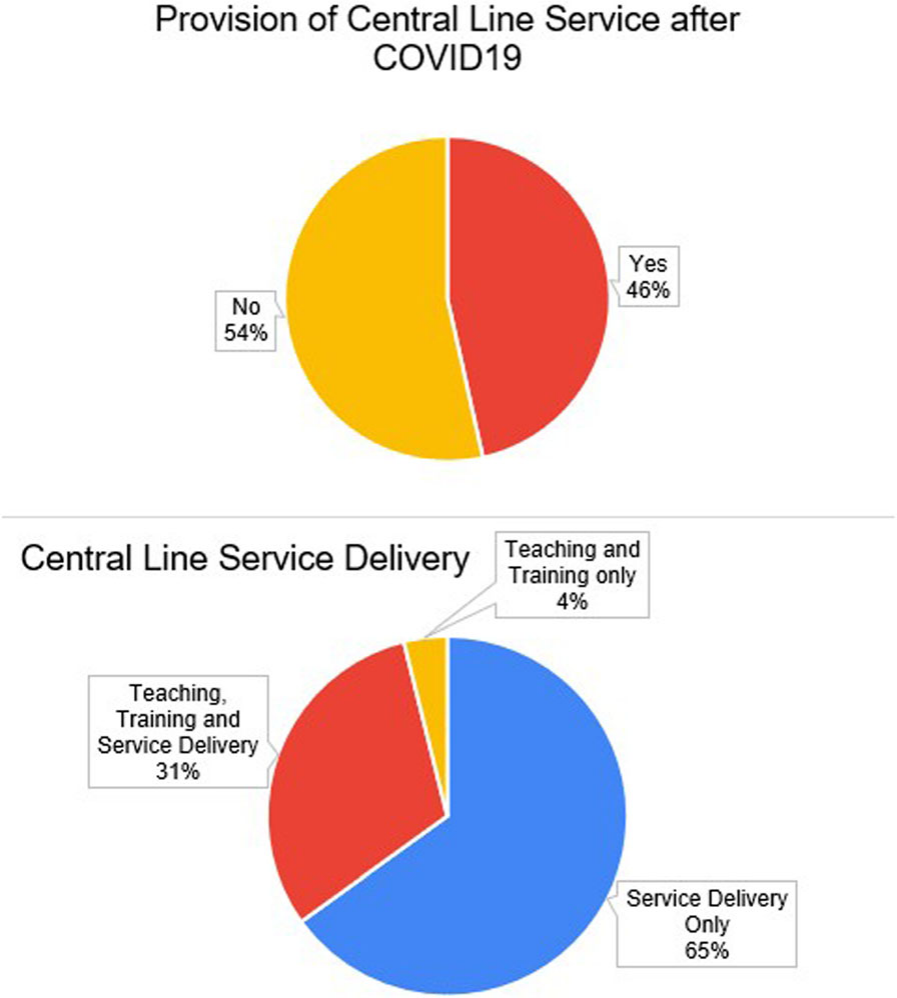
Provisions of central line service in response to COVID-19

In response to the COVID-19 pandemic, 81% of respondents were still able to provide a 24/7 on call service. Only a small number of respondents (1.3%, 3 out of 228) were unable to offer 24/7 on-call service despite previously offering this service. Fifty nine percent however, reported making changes to their normal day to day practice to allow the 24/7 service to continue during the COVID-19 crisis. Eighty one percent of those who reported changes to day-to-day practice reported reduced diagnostic radiology activity and 36% reported increased working hours. Only 5% had been required to reduce out-of-hours cover and none had needed to cease all out of hours IR activity. Fifty four percent are now involved in the provision of a central venous catheter service for patients suffering with COVID-19 infection (Fig. 4). Most respondents provided the service directly, whereas a smaller proportion 36% have been involved in training teaching and training of other staff members as well as line insertion itself. Several respondents offered the service to Intensive Care departments and or Nightingale temporary field hospitals, but the latter facility had not been utilized at the time of the survey.

Of those questioned, 91% are still offering endovascular services and 98% of Genitourinary IR services. Similarly, 92% of respondents have continued providing HPB services. Table 1 outlines the changes in service delivery described by survey respondents. Several respondents commented that they had witnessed a reduced demand for elective IR procedures in general (Table 1).

**Table 1:** Service provision in IR in the COVID-19 pandemic

Service	No reduction in service	25% reduction in service	50% reduction in service	> 75% reduction in service
**Endovascular**	7.5% (16)	13.1% (28)	32.7% (70)	46.7% (100)
**Hepatobiliary**	37% (77)	15.9% (33)	26% (54)	21.2% (44)
**Genitourinary**	27.7% (61)	25.9% (57)	30.5% (67)	15.9% (35)

Regarding training, 38% of respondents provided IR trainees with additional training material during this crisis either in on-line learning material or with practical hands on training.

## Discussion

The effect of COVID-19 is likely the greatest challenge that the NHS has witnessed to date (Rasanathan and Nolan 2020). Along with other departments, the radiology and Interventional Radiology departments have been required to rapidly adapt their services to this evolving crisis. In line with public health directives, UK IR departments have made extensive changes but continue to deliver safe urgent and emergent care for positive as well as negative COVID-19 patients (BSIR 2020). It is advisable to have a designated IR suite for COVID-infected patients and detailed patient flow pathways created to enable safe transfer of patients to and from the IR suite from other hospital departments (Tsou et al. 2020; Too et al. 2020).

This survey confirms that IR departments have continued to provide essential on-call and emergent services during this crisis with only a small percentage reducing out of hours cover in response to the crisis. They have also demonstrated rapid response and flexibility, with over a third of respondents making changes to their day to day activity to continue to provide seven-day IR cover. The IR community should be commended for its collaboration with frontline departments already under tremendous pressure by assisting with the provision of a central line service to Intensive Care Units.

Reassuringly, we have demonstrated that most IR services have been maintained during the pandemic thus far, and almost no centre has completely ceased service delivery. Advantages unique to the IR specialty may have enabled maintenance of the service and these include access to a day case unit, local anaesthetic procedures with only minimal reliance on anaesthetic support and small numbers of aerosolizing procedures. Several respondents commented that the tumor ablation service had been negatively impacted by the wider pressures on the anaesthetic service.

Approximately one third of respondents reported no reduction in HPB or GU services. This maintained demand may be accounted for by a reduction of endoscopic or urological services perhaps due to aerosolizing risk or reduced anaesthetic support. The described reduction in services by others, may be due to a decreased demand for Interventional Radiology, perhaps because of reduced emergency presentations and cancellation of elective surgery within the hospital. The recent reports of a 63% reduction in emergency hospital admissions in comparison to the previous year would support the notion that the “stay at home” message is resulting in a significant reduction in the trauma workload (Stephens and Pritchard 2020).

The pandemic has unfortunately had a significant impact on Specialty Trainees and IR Fellows. Cancellation of postgraduate exams, including the FRCR, EBIR, all conferences and face-to-face courses will undoubtedly dilute the value of this time in training and may impact upon trainees’ progression. Although approximately one third of respondents have provided their trainees with additional learning material, it is apparent that IR trainees will need a thoughtful plan to address the deficiencies and their training needs. Given that this crisis is likely to persist for many months, it is essential that relevant educational material is delivered urgently to allow continued professional development for IR trainees and fellows during this time, to avoid unnecessary delays in completion of training, and to ensure that they remain ready to take on the role of a consultant.

As we write, the COVID-19 pandemic has likely peaked in the United Kingdom and we now enter Phase II of the NHS Response to COVID-19 (Stephens and Pritchard 2020). With the mandate to increase non-COVID19 urgent services over the next 6 weeks it is vital that this restoration is managed effectively (Stephens and Pritchard 2020). Recommencement of other elective services within hospitals, is likely to result in increased demand for IR procedures again, possibly with a significant rebound excess, which may result in significant stresses to a workforce previously under pressure. Such pressures may be significant, particularly as whilst there remains no vaccine against COVID-19, some staff (IR, Radiographers and Nurses) may need to remain in a shielded environment.

Our response requires robust and clear guidance from IR societies and the Royal College of Radiologists to help IR departments deliver a safe service for patients’ and the IR workforce over the coming year(s). As other specialties have outlined, it will be important to have a phased reintroduction of services (Penman et al. 2020; RCR 2020). his requires clear pathways to separate COVID-19 positive and negative patients into different treatment zones. Meticulous pre-planning with careful review and prioritization of requests (Royal College of Surgeons England and Royal College of Surgeons Glasgow 2020), telephone screening of patients prior to elective procedures. In support of this, rapid antibody testing availability will be needed (Penman et al. 2020). For the procedures themselves, we must expect slower throughput of cases with allowance for enhanced safety procedures and appropriate equipment cleaning after individual cases. This survey showed that the overwhelming majority of respondents have access to a day case facility and this, at least in part, enabled services to continue despite severe shortage availability of hospitals beds. The safety and cost effectiveness of day case IR services have long been established (Huang et al. 2008; Macdonald et al. 2002). It is now time, more than ever, that IR management should engage further with health care provider authorities to invest more in these types of services.

It is uncertain for what length of time we will feel the impact of COVID-19 on our day to day practice.

## Conclusion

The results of this survey have demonstrated that, as a modern progressive specialty, interventional radiology adapted quickly to meet the demands of this unexpected healthcare crisis, and that it is, and remains, an essential service to a wide variety of hospital departments. The ability to deliver minimally invasive therapy will be considerably facilitated by the support and development of separate day case facilities which are appropriately staffed by IR nurses and doctors. Such ongoing investment will facilitate safe practice and cost-effective delivery of IR during “ordinary” as well as crisis times.

## Supplementary information

**Additional file 1.**

## Data Availability

The datasets used and/or analysed during the current study are available from the corresponding author on reasonable request.

## Abbreviations

IR: Interventional Radiology
NHS: National Health Service
HPB: Hepatobiliary
BSIR: British Society of Interventional Radiology
