# Supplementary figures and images for: Response of UK interventional radiologists to the COVID-19 pandemic – survey findings
Source: CVIR Endovasc. 2020 Jun 26;3:41. doi: 10.1186/s42155-020-00133-2 (PMC7317887; doi:10.1186/s42155-020-00133-2)

Appendix
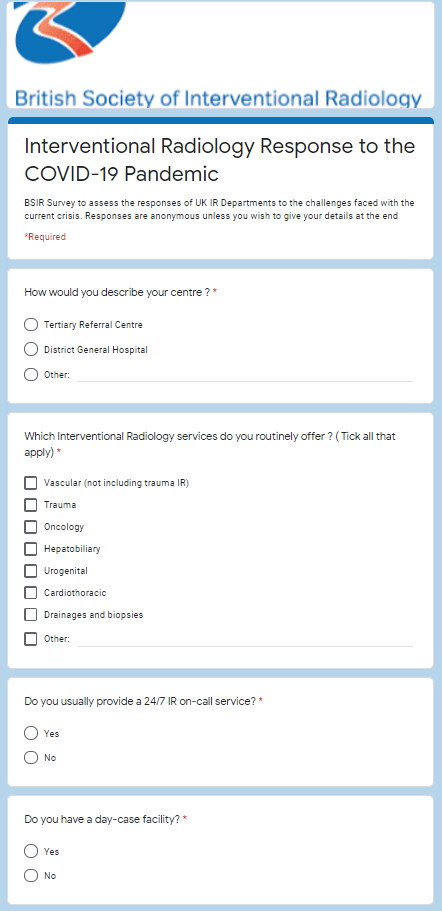
:


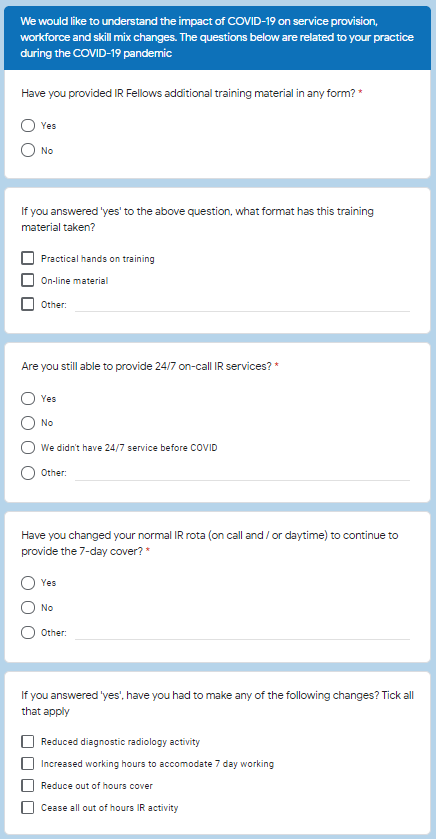

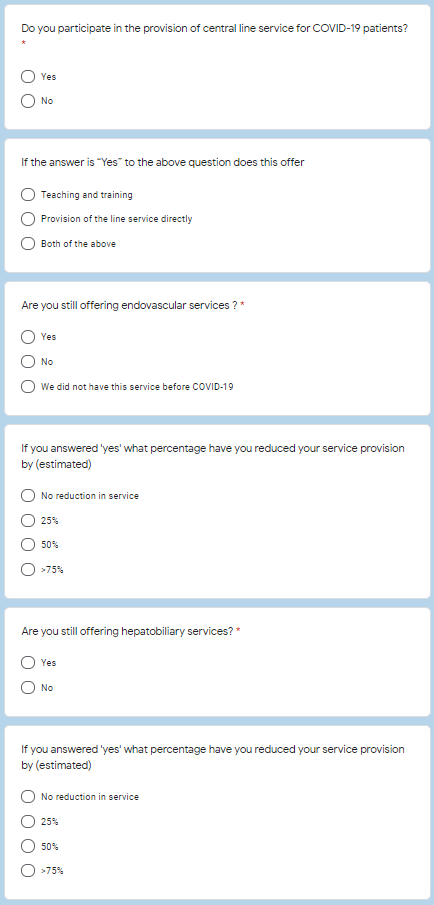

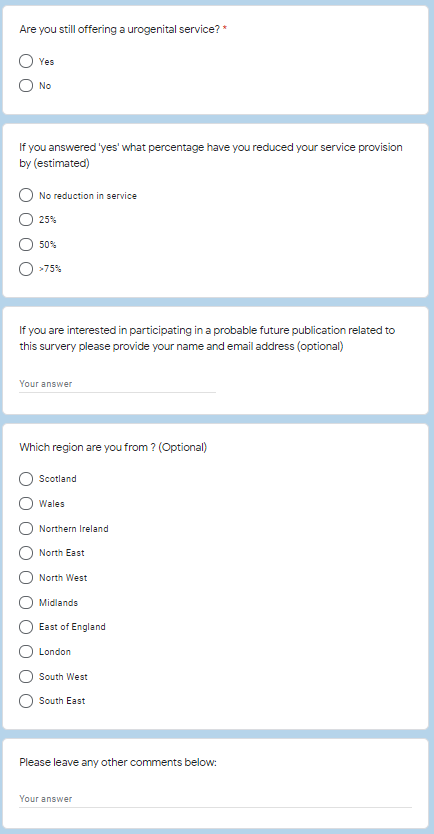

Supplement: Supplementary file 1 — Additional file 1. [file 42155_2020_133_MOESM1_ESM.docx]
